# Application of basic and composite thrombelastography parameters in monitoring of the antithrombotic effect of the low molecular weight heparin dalteparin: an in vivo study

**DOI:** 10.1186/1477-9560-7-14

**Published:** 2009-11-10

**Authors:** Ramin Artang, Niels J Frandsen, Jørn Dalsgaard Nielsen

**Affiliations:** 1 Coagulation Laboratory, Gentofte University Hospital, Hellerup, Denmark; 2 Mercy Heart Center, Sioux City, Iowa, USA; 3 Department of Cardiology, Gentofte University Hospital, Hellerup, Denmark

## Abstract

**Background:**

Low molecular weight heparin (LMWH) is in vast usage for treatment of thromboembolic diseases such as deep venous thrombosis and acute coronary syndromes. There are certain clinical situations where a quick point of care testing of the effect of LMWH would be useful. At this point there are no point of care devices available in the market for monitoring the effect of LMWH. Thrombelastography (TEG) evaluates the viscoelastic properties of blood during coagulation. The clinical application of TEG in monitoring LMWH treatment is not yet well defined. The purpose of this in vivo study was to systematically evaluate the most suitable TEG parameters for evaluation of the antithrombotic effect of LMWH. We furthermore evaluated for the first time the usefulness of the composite TEG parameter the Thrombodynamic Ratio (TDR) in monitoring LMWH treatment.

**Methods:**

Healthy male volunteers (n = 7) were injected subcutaneously with the LMWH dalteparin 120 IU/kg. TEG parameters and antifactor Xa levels were measures at baseline, 2, 4, 5 and 24 hours after the injection. Correlation between TEG parameters and antiXa were calculated. The sensitivity and specificity of the TEG parameters for plasma levels of antiXa in the therapeutic range of 0.5 - 1.0 U/ml were calculated.

**Results:**

All basic TEG parameters correlated significantly with antiXa levels. Among the basic parameters, the TEG reaction time R had the best correlation with antiXa levels with the most favorable combination of sensitivity and specificity for the therapeutic range of antiXa levels (r = 0.82, p < 0.0001, sensitivity 68%, specificity 100%). The composite TEG parameter TDR demonstrated the best correlation with antiXa levels, and an even more favorable combination of sensitivity and specificity compared to any of the basic parameters (r = - 0.87, p < 0.0001, sensitivity 95%, specificity 79%).

**Conclusion:**

The TEG reaction time R and TDR are the most suitable TEG parameters for evaluation of the antithrombotic effect of dalteparin with a highly significant correlation with antiXa levels in healthy male volunteers. Measures for uniform clinical use of these parameters are proposed. Larger clinical trials are needed to correlate R and TDR with clinical outcomes.

## Introduction

Application of low molecular weight heparin (LMWH) in the treatment of acute coronary syndromes and deep venous thrombosis has been evaluated in several large-scale trials [1-5]. The main advantage of the LMWH as compared to the unfractionated heparin (UFH) is that the LMWH can be administered in weight adjusted subcutaneous doses without the need for monitoring coagulation parameters. There are, however, a number of circumstances where monitoring the effect of LMWH on the coagulation system is desirable. These conditions include bleeding situations, patients with renal failure, children and patients with high or low body weight. In addition preterm pregnant women with thrombophilia who are on LMWH treatment constitute a risk of bleeding complications during regional anesthesia or surgical delivery [6,7]. The conventional test for monitoring the LMWH function is anti-factor Xa activity level (antiXa) in plasma. The antiXa measurement is however both time and labor intensive and therefore not suited routine clinical application. A point of care test reflecting the antithrombotic effect of LMWH will therefore fulfill an important clinical function [6]. At this point there are no point of care devices available in the market for monitoring the effect of LMWH.

Thrombelastography (TEG) is a point-of-care test for evaluation of hemostasis. It measures the viscoelastic properties of clotting blood. During the *ex vivo *clotting process, changes in viscoelastic properties are dependent on the amount and interaction of platelets and fibrinogen, as well as the fibrin formation rate by thrombin [8]. Earlier studies have shown correlation between TEG variables and routine coagulation tests (platelet count, prothrombin time, activated partial thromboplastin time, antithrombin and fibrinogen) [9,10]. There is extensive experience with TEG in monitoring hemostasis on patients undergoing orthotopic liver transplantation and open heart surgery where it is proven to be superior to routine coagulation tests in predicting peri- and postoperative bleeding and use of blood products [11,12]. Monitoring unfractionated heparin therapy by TEG has also been described [13]. There are however only a few in vivo studies available on monitoring LMWH therapy by TEG in humans [14-17]. The clinical application of TEG in monitoring LMWH treatment is not yet well defined. While LMWH seems to alter all TEG parameters, the TEG reaction time has been the parameter most investigated. Whether the reaction time is the most suited parameter for monitoring LMWH treatment has not been systematically and adequately evaluated. Furthermore, there has so far been no attempt on evaluation of a single integrated TEG parameter consisting of several basic parameters for assessment of LMWH treatment. We have previously introduced the Thrombodynamic Ratio (TDR), a composite TEG parameter [18]. The purpose of this in vivo study was to systematically examine the correlation between the AntiXa and the basic TEG parameters. We furthermore sought for the first time to evaluate the usefulness of TDR in monitoring LMWH treatment. In addition measures for uniform clinical use of TEG parameters are proposed.

## Materials and methods

This study was approved by the Regional Review Board of Copenhagen County, Denmark. Informed consent was obtained before inclusion.

### Thromboelastography

The principles of the thromboelastographic measurement have previously been described [8,9]. The basic TEG parameters include reaction time R, angle of alpha (α) and Maximum Amplitude (MA). Reaction time is prolonged by clotting factor deficiencies, warfarin, unfractionated heparin and LMWH treatment [13,19]. Aspirin has no effect on reaction time [20]. In the TEG literature, the alteration of R value from baseline after the LMWH injection is defined as ΔR. Thrombodynamic Ratio (TDR)(18) is a composite TEG parameter defined as:



TDR has no units. As previously described the thrombophilic conditions have a tendency to increase MA and α, and decrease R [21]. In contrast antithrombotic treatment such as thrombolytics, and thrombin inhibitors tend to decrease MA and α, and increase R. The TDR in essence portrays an amplified profile of the prothrombotic or antithrombotic state in the sample. As a consequence a large TDR value indicates a prothrombotic condition and a small TDR indicates an antithrombotic condition in the sample. We used a Thrombelastograph^® ^coagulation analyzer 5000C (Haemoscope Corp, Niles, IL, USA). Celite activated TEG analysis was performed on 330 μl citrated whole blood that was recalcified with 30 μl 0.2 M CaCl_2_. The parameters R, α and MA were measured and the average of the duplicate levels was recorded for the statistical analysis. The TDR was calculated based on the average of the duplicate levels of the basic parameters and applied in the statistical analysis. All TEG analyzes were performed within one hour after sample collection.

### Blood sampling

Healthy male volunteers were treated with a single subcutaneous injection of dalteparin. No other antithrombotic agents, including aspirin or non-steroidal anti-inflammatory agents, were allowed a week prior to inclusion in the study. The blood samples were collected just before the injection of dalteparin as baseline values. After the subcutaneous injection of dalteparin sodium 120 IU/kg, (Fragmin^®^, Pfizer, New York USA), blood samples were collected at 2, 4, 5 and 24 hours. Samples were analyzed for antiXa level and the TEG parameters.

### Anti-factor Xa analysis

For AntiXa analysis citrated blood was centrifuged at 2000 g for 10 minutes. All plasma samples were stored at -80°C and then analyzed in batch. AntiXa was measured with Chromogenic assay on STA-R^® ^apparatus (Diagnostica Stago, France). The coefficient of variation was 2%.

### Statistical Analysis

All data are presented as mean and standard deviation (SD) unless otherwise indicated. Spearman correlation coefficient (r) was calculated for correlation between the TEG parameters and antiXa. P values less than 0.05 (two tailed) were considered statistically significant. The sensitivity and specificity of each TEG parameter for plasma levels of antiXa in the therapeutic range of 0.5 - 1.0 U/ml were calculated. The sensitivity was defined as number of the true positives divided by the sum of true positives and false negatives. The specificity was defined as number of the true negatives divided by the sum of true negatives and false positives. The cut off point was chosen as the mean value of each parameter obtained from a sample of 49 healthy donors. These values were compatible with the reference range provided by the manufacturer. Statistical analyses were performed using Prism software version 5.0 (GraphPad Software Inc. La Jolla CA, USA) and MedCalc version 10.3 (MedCalc Software, Mariakerke Belgium).

## Results

**Figure 1:**
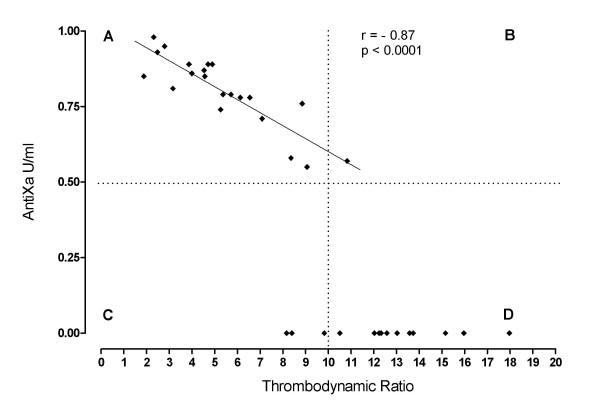
**Correlation between Thrombodynamic Ratio (TDR) and AntiXa levels is demonstrated**. The sensitivity and specificity of TDR for plasma levels of antiXa in the therapeutic range of 0.5 - 1.0 U/ml (horizontal dotted line) were calculated using cut off point of 10 (vertical dotted line). The dots in the left upper quadrant (A) represent TDR values less than 10 that correspond to therapeutic range of AntiXa. Dots in the right upper quadrant (B) are TDR values more than 10 that correspond to therapeutic range of AntiXa. Dots in the left lower quadrant (C) are TDR values less than 10 that correspond to non therapeutic AntiXa and dots in the right lower quadrant (D) represent TDR values more than 10 that correspond to non therapeutic AntiXa. The sensitivity was defined as number dots in A/A+C = 20/20+1 ≈ 0.95. The specificity was defined as number of dots in D/D+B = 11/11+3 ≈ 0.79.

**Figure 2:**
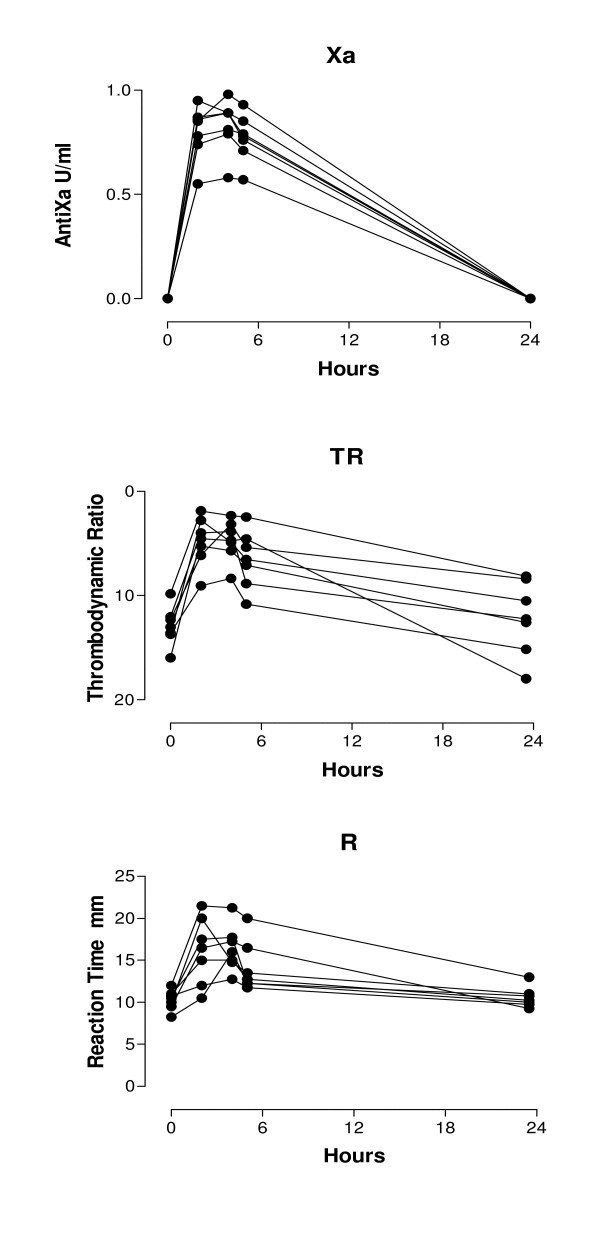
**Anti-factor Xa (top), Thrombodynamic Ratio (middle) and Reaction time (bottom) levels plotted parallel at 2, 4, 5 and 24 hours after injection of single subcutaneous injection of 120 IU/kg of dalteparin in seven healthy male volunteers**. Circles represent individual subjects.

Seven healthy volunteers (median age 36, total range 28-56) were included. A significant correlation was observed between antifactor Xa and all 3 basic TEG parameters based on 21 pairs of data (Table 1). The levels at baseline and 24 hours where the antiXa levels were zero were excluded from the correlation analysis. A highly significant correlation between TDR and AntiXa was observed (r = - 0.87, p < 0.0001) (Figure 1). The sensitivity and specificity of each TEG parameter and TDR for plasma levels of antiXa in the therapeutic range was calculated using the cut off point of 13 mm for R, 66° for alpha, 58 mm for MA and 10 for TDR (Figure 1). The R values above 13 mm had the highest specificity for therapeutic range of antiXa levels. The MA values less than 58 mm and alpha less then 66° had the highest sensitivity for therapeutic range of antiXa levels with poor specificity. TDR levels less than 10 had the highest combination of the sensitivity and specificity for therapeutic range of antiXa levels. Plotting the individual values over 24 hours for each subject after dalteparin injection showed homogenous response measured by antiXa level, R and TDR (Figure 2).

**Table 1:** Correlation coefficients of TEG parameters compared to anti-factor Xa.

	**Correlation Coefficient (r)**	**Sensitivity (%)**	**Specificity (%)**	**P value**
**R**	0.82	68	100	<0.0001
**α**	- 0.72	100	57	<0.0001
**MA**	- 0.68	100	36	<0.0001
**TDR**	- 0.87	95	79	<0.0001

Sensitivity and specificity of the TEG parameters for anti-factor Xa level in therapeutic range of 0.5 - 1.0 U/ml.

α: angle of Alpha, MA: maximum amplitude; R: reaction time, TDR: thrombodynamic ratio.

## Discussion

In this study we have demonstrated a strong correlation between all basic TEG parameters and antiXa levels in healthy volunteers receiving a single dose subcutaneous injection of dalteparin. We furthermore demonstrated that the Reaction time R had the overall most favorable combination of correlation coefficient, sensitivity and specificity for clinical use among the basic TEG parameters. This finding demonstrated that R is the most suitable basic parameter for monitoring LMWH treatment. In the present study we demonstrated for the first time that the composite TEG parameter TDR has a highly significant correlation with AntiXa levels. It in fact has an even more favorable combination of sensitivity and specificity for the therapeutic range of antiXa levels than any of the basic TEG parameters. The application of a composite TEG parameter for monitoring unfractionated heparin has been attempted in the past [13]. The composite parameter used was termed Thrombodynamic Potential Index (TPI) defined as: MA • 100 (100 - MA)/2 • K. The other known composite TEG parameter is Clotting Index (CI) defined as: CI = -0.6516R - 0.3772K + 0.1224MA + 0.0759α - 7.7922. The limitation of both of the above mentioned factors is the parameter K. The Clotting time K was among the original TEG parameters developed by Hartert and is defined as distance or time from the 1 mm wide point on TEG profile to the 20 mm wide point [8]. In the modern antithrombotic treatment that includes single agents or combination of agents such as, tissue plasminogen activators, glycoprotein IIb/IIIa inhibitors, thienopyridines, thrombin inhibitors and selective factor X inhibitors it is possible that due to antithrombotic action in vivo, the TEG profile would never reach the wide point of 20 mm making the above mentioned formulas useless. With this in mind the TDR was developed, so that it is independent of the parameter K. The clotting dynamics that K represents are also reflected in the angle of α. Since not only R but all the basic parameters were altered significantly by dalteparin, it seemed intuitive that a composite parameter such as TDR would correlate strongly with antiXa levels. To our knowledge this is the first report of the application of a composite TEG parameter for assessment of LMWH treatment. The advantage of the composite TEG parameter TDR is that it incorporates all the basic TEG parameters provides a more user friendly approach to the concept of TEG from a clinical standpoint. It is easier for the clinician to consider a single parameter rather than 3 or 4 parameters. The disadvantage of such composite parameter is that it may diminish subtle changes measured in only one of the parameters. In addition as seen in figure 2, due to the mathematical composition of TDR, the coefficient of variation is larger as compared to the basic TEG parameters.

There has been only one previously published in vivo study on the application of TEG in monitoring dalteparin therapy in humans. Shinoda and coworkers studied 28 patients with kidney failure who were monitored during hemodialysis after receiving dalteparin [14]. The study showed a weak correlation between reaction time and antiXa (r = 0.402, p < 0.05). The timing of the sample collection from the dalteparin injection was not specified and the other TEG parameters were not analyzed. The samples were obtained from the dialyzer during extracorporeal blood circulation that may have had an impact on the results. Besides the case reports and the study by Shinoda, we indentified only 3 other in vivo studies in the literature regarding the application of TEG for monitoring LMWH treatment in humans. Out of the 3 studies, the only study with appropriate dosage and timing of the sample collection that provided calculation of correlation coefficient was by Carroll and co-workers, who examined 15 pregnant subjects with thrombophilia after treatment with subcutaneous enoxaparin [17]. The parameter applied in the latter study was ΔR with a correlation coefficient of 0.90 comparable to the correlation found in the present study and confirming the suitability of the TEG parameters such as R or ΔR for such purpose. The other TEG parameters were not specified. One of the limitations of solely applying ΔR for monitoring LMWH treatment is, that if the clinical situation does not allow a baseline measurement of R then ΔR cannot be calculated and the clinician is then left with a patient already injected with LMWH and is interested in knowing the degree of anticoagulation for bleeding risk assessment prior to a procedure. For this reason, we propose that an absolute R value above 13 mm is highly specific and absolute TDR value less than 10 is highly sensitive for therapeutic range of AntiXa levels.

### Limitations

This study was limited due to small sample size. Furthermore this in vivo study was on healthy male volunteers treated with dalteparin monotherapy. Since the dose of dalteparin applied in this study was according to established guidelines by the manufacturer, none of the subjects reached supra-therapeutic levels of antiXa. In addition none of the subjects had sub-therapeutic levels of antiXa in the time intervals chosen in this study. The correlation of TEG parameters with antiXa levels above 1 U/ml and below 0.5 U/ml were therefore not evaluated and may need further investigation. These findings need to be verified with the other LMWH's and co-medication with other antithrombotic agents in patients of both gender with various disease states, before a more general usage of the method can be recommended.

## Conclusion

Based on our observations, the TEG reaction time R and the composite parameter of TDR have a substantial correlation with anti-factor Xa levels in healthy volunteers. In order to investigate the value and usefulness of TEG in predicting the bleeding or thrombotic complications, larger trials on patients treated with different antithrombotic agents including different LMWH preparations are needed to correlate abnormal R and TDR values with negative clinical outcomes. The current study may be an important first step.

## Abbreviations

AntiXa: Anti-factor Xa; α: angle of Alpha; CI: Clotting index; K: Clotting time; ΔR: The alteration of R value from baseline after dalteparin injection; MA: maximum amplitude; R: reaction time; TAN: trigonometric function, tangent of an angle; TDR: thrombodynamic ratio; TEG: thrombelastography; TPI: thrombodynamic potential index.

## Competing interests

The authors declare that they have no competing interests.

## Authors' contributions

RA was responsible for the conceptualizing and the design of the study, blood sample collections, TEG measurements, the statistical analysis and preparation and submission of the manuscript. NJF was involved in the design and conceptualizing of the study and had a substantial contribution in finalizing of the manuscript. JDN was the senior investigator, responsible for conceptualizing and the design of the study and had substantial contribution in finalizing the manuscript. All authors read and approved the final manuscript.
